# Enhanced toughness and thermal conductivity for epoxy resin with a core–shell structured polyacrylic modifier and modified boron nitride

**DOI:** 10.1039/c8ra10645b

**Published:** 2019-03-15

**Authors:** Chen Xu, Taoguang Qu, Xiaojie Zhang, Xiongwei Qu, Nongyue Wang, Qingxin Zhang, Beckry Abdel-Magid, Guohua Li

**Affiliations:** Institute of Polymer Science and Engineering, School of Chemical Engineering, Hebei University of Technology Tianjin 300130 P. R. China xwqu@hebut.edu.cn bamagid@winona.edu nkligh@126.com; College of Materials Science and Engineering, Beijing University of Chemical Technology Beijing 100029 P. R. China; Department of Composite Materials Engineering, Winona State University Winona MN 55987 USA

## Abstract

A new epoxy-based composite with higher toughness and thermal conductivity was developed. First, a poly(*n*-butyl acrylate)/poly(methyl methacrylate-*co*-glycidyl methacrylate) (PBMG) core–shell structured latex was prepared by seeded emulsion polymerization to toughen the epoxy resin (EP). Second, boron nitride particles were modified into nano-scale sheets and added to the epoxy/PBMG blend to improve the thermal conductivity of the resulting composite material. The properties of the constituent materials were determined prior to fabrication and testing of the composite product. The monomer conversion in the emulsion polymerization process of the PBMG was checked by determining the solid particle content. The PBMG particle size was characterized by dynamic laser scattering, and the morphology of the particles was characterized by scanning and transmission electron microscopy. The exfoliation of the modified boron nitride (MBN) flakes was verified by TEM and Raman microscopy. The mechanical properties and the thermal conductivity of the EP/PBMG/MBN composite were determined at various constituent contents. Results showed that the unnotched impact strength of the composite increased by 147%, the flexural strength increased by 49.1%, and the thermal conductivity increased by 98% compared with pristine EP at a PBMG content of 5 wt% and MBN content of 7 wt%. With the enhanced properties and ease of fabrication, the developed composite has good potential for application in high-end industries such as microelectronics packaging.

## Abstract 2

A ternary composite is fabricated with exfoliated boron nitride, core–shell polyacrylic, and epoxy. The mechanical properties and the thermal conductivity were increased synergistically, allowing the composite to be used as the capsulations.

## Graphical abstract

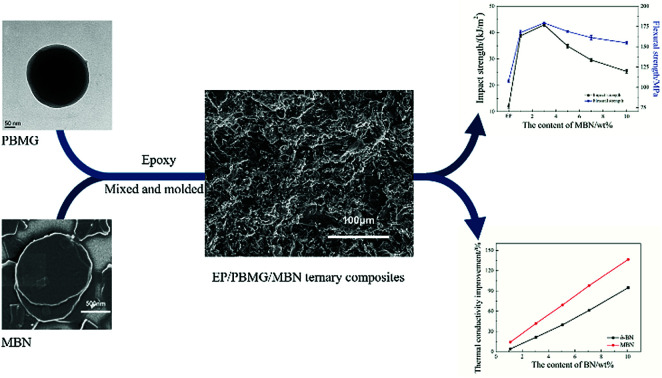

## 1. Introduction

Epoxy resin (EP) has been widely used due to its many desirable properties such as: good electrical insulation, heat resistance, ease of processing, and low cost. It has been used in composite laminate, adhesives, semiconductor packaging, aerospace, and other applications.^1–5^ However, the cured resin has high cross-linking density and is very brittle with low resistance to the initiation and growth of cracks, which make the epoxy matrix undesirable in many applications. Researchers have been working to toughen epoxy and broaden its use in more demanding applications. To achieve this purpose, a variety of reinforcements, including liquid rubber tougheners,^6–8^ core–shell rubber (CSR) particles,^9–19^ thermoplastics,^20,21^ and inorganic filler^22–24^ have been used. However, sometimes these led to a decrease in glass transition temperature, modulus and tensile strength of the epoxy matrix. To overcome this conflict, hybrid epoxy composites reinforced by both rigid particles and rubber particles have been developed by incorporating the two reinforcing agents,^25–29^ which are shown to possess a synergetic toughening effect. Acrylic core–shell polymers (CSP) have been used in toughening various resins because of their good weather resistance, low shrinkage, and low cost of preparation. Moreover, the composition and size of the particles can be controlled to obtain desired properties. These particles consist of a soft rubbery core inside a harder polymer shell. The particle size can be controlled as they are formed by emulsion polymerization before they are dispersed into the epoxy resin.^30–34^

With the rapid development in electronic technology, demand for high performance and small size electronic devices has been on the rise. Such high demand causes these devices to generate and accumulate a great amount of heat that should be effectively dissipated to prolong the service life of the devices.^35^ Commonly suggested fillers to dissipate the heat include: graphite,^36^ carbon nanotubes^37,38^ and graphene,^39–41^ however these fillers are also electrically conductive and cannot be applied in composite materials that are used for electrical insulation. To effectively solve the thermal dissipation problem and obtain high performance, different fillers have been introduced into polymers to provide thermally conductive but electrically insulative polymer-matrix composites. These fillers include oxides (Al_2_O_3_,^42^ SiO_2_,^43^ and ZnO^44^), diamond,^45^ fly ash^46^ and nitride (AlN and BN)^47,48^ which are thermally conductive but electrically insulative. Hexagonal boron nitride (h-BN) represents a filler with a thermal conductivity of up to 600 W (m^−1^ K^−1^) and high electrical insulation^49^ that can be used as a material to dissipate heat in such applications.^50–57^ Researchers have shown that functionalized h-BN has high thermal conductivity, excellent thermal properties, and that it can be incorporated as a conductive filler in polymeric matrices.^58–64^ Gu *et al.* used NH_2_-POSS functionalized nBN fillers to improve the thermal conductivities for PPS dielectric nanocomposites.^58^ However, research on improving both the thermal conductivity and toughness of epoxy matrix is scarce and not widely reported. A novel high performance composite material consisting of epoxy, core–shell polymers, and modified boron nitride developed to improve both of these properties, is presented in this paper. The composite has been successfully prepared by sequentially adding moderate amounts of poly(*n*-butyl acrylate)/poly(methyl methacrylate-*co*-glycidyl methacrylate) core–shell structured particles (PBMG) and modified boron nitride (MBN) to the epoxy (EP) resin.

## 2. Experimentation

### 2.1 Materials

The constituent materials used to develop the epoxy-based composite include: epoxy resin (E51) from Nantong Xingchen Synthetic Materials Co.; methyl tetrahydrophthalic anhydride (MTA) and 2,4,6-tris(dimethylaminomethyl)phenol (DMP-30) from Puyang Huicheng Electronic Materials Co.; *n*-butyl acrylate (BA), methyl methacrylate (MMA), and allyl methacrylate (ALMA) from Tianjin Tianchen Chemicals Industry Co.; potassium persulfate (KPS) from Tianjin Hongyan Chemical Reagent Co.; 1,4-butanediol diacrylate (BDDA) obtained from Tianjin Chemical Co.; glycidyl methacrylate (GMA) from Tianjin Chemical Reagent Research Institute; and anionic surfactant, sodium bis(2-ethylhexyl)sulfosuccinate from Tianjin Reagents Co. The hexagonal boron nitride (h-BN) was obtained from Qingzhou Matt Kechuang Materials Co.; the benzyl benzoate was purchased from Aladdin; and the absolute alcohol was supplied by Tianjin Fuchen Chemical Reagent Factory. All the chemicals were used as received without further purification. Deionized water (DIW) was used in all the polymerization processes.

### 2.2 Emulsion polymerization process of PBMG

Poly(*n*-butyl acrylate)/poly(methyl methacrylate-*co*-glycidyl methacrylate) (PBMG) latex was synthesized in a 500 ml four-necked flask equipped with a mechanical stirrer and reflux condenser. The temperature of the water bath was maintained at 78 °C, and the reaction was carried out in an argon atmosphere. First, the surfactant (0.25 g) and deionized water (140 g) were added to a four-necked flask and stirred at a 180 rpm for 30 minutes. Then a mixture of seed monomer (BA, 10 g) and a crosslinking agent (ALMA, 0.054 g) were added to the above dispersed system. After stirring for 10 minutes, a KPS aqueous solution (KPS, 0.43 g per DIW, 20 g) was added to start the polymerization reaction. More KPS aqueous solution (0.11 g/10 g) was added after 55 minutes and stirred in for 5 minutes to complete the reaction of the seed stage. The second stage was the growth stage, which involved two layers of pre-emulsified monomers. The first layer was the rubber core, containing BA (130 g), surfactant (1.625 g) and ALMA (2.275 g). The second layer was the rigid shell, consisting of MMA (60 g), surfactant (1.2 g), and functional monomer GMA (1.5 g). The pre-emulsified mixture was added into the reaction flask at a rate of 2 ml min^−1^ during the growth stage. The initiator, KPS aqueous solution (0.043 g/10 g), was added into the reaction flask at 60 min intervals after the beginning of the second stage.

**Fig. 1:**
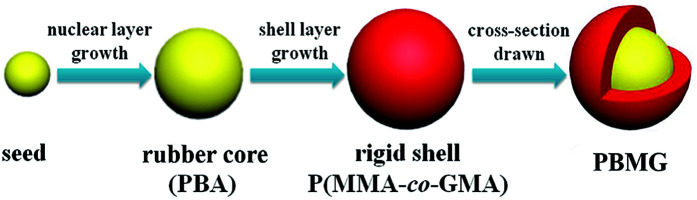
Preparation procedure of PBMG.

The final mixture was allowed to remain for another 60 minutes to ensure that the monomer reaction was complete. Then the latex was cooled to room temperature and filtered through a 53 μm sieve to obtain the coagulated content. Finally, the PBMG particles were obtained after freezing the filtrate for 12 h and freeze-drying it for another 12 h. The product was collected and filtered through a 100 mesh screen to obtain the final product. A schematic diagram of the preparation of PBMG is shown in Fig. 1.

### 2.3 Characterization of the PBMG latex particles

At 30 min intervals, 5 ml samples of the latex were transferred into pre-weighed vials containing 1 ml hydroquinone solution (2 wt%) and then cooled down in an ice batch to terminate the polymerization. Gravimetric analysis was used to determine the monomer conversion. The particle size and polydispersity index (PDI) were measured with dynamic light scattering (DLS) using a Malvern Zetasizer NANO-ZS90 (Worcestershire, UK). The cell temperature was held at 25 ± 0.1 °C, and the *z*-average diameter was calculated.

The growth of the latex particle size in the seed emulsion polymerization was investigated. The particles diameters determined by DLS were compared with theoretical particle diameters which were calculated according to the following equation:^65^

1 *d*_*t*_ = (*M*_*t*_*I*_*t*_/*M*_s_)^1/3^ × *d*_s_

where *d*_*t*_ is the diameter of the particle at time *t*, *M*_*t*_ is the total mass of the monomers added at time *t*, *I*_*t*_ is the instantaneous conversion at time *t*, *M*_s_ is mass of monomer added in the seed stage, and *d*_s_ is the seed particle diameter as measured by DLS.

Instantaneous and overall conversions were calculated using the following equations:^66^

2 Instantaneous conversion% = [mass of polymer formed/mass of monomer added] × 100

where the mass of monomer added is the sum of the monomer at the seed stage and any monomer that has been added at each sampling time during the growth stage.

3 Overall conversion% = [mass of polymer formed/total mass of monomer] × 100

where the total mass of monomers added is the sum of monomers at the seed stage and all monomers added during the growth stage.

### 2.4 Morphology of the PBMG core–shell particles

The morphology of the PBMG latex was characterized by a JEOL JEM-2100 transmission electron microscope (TEM). The latex was first dispersed in deionized water with ultrasonic waves for 3 min, then single drops of diluted solution were deposited onto a carbon-coated copper grid, and dried with infrared light to prepare samples for TEM analysis.

The external morphology of the latex particles was characterized by an FEI Nova NanoSEM 450 field emission scanning electron microscope (SEM). The sample was diluted with distilled water to achieve the desired concentration, followed by ultrasound treatment for 5 min, then single drops of diluted solution were deposited on a silicon wafer which was then placed in a vacuum and dried at room temperature for 24 h. The surface morphology of the core–shell particles was observed and characterized.

### 2.5 Modification of the BN powder

The preparation of boron nitride nanosheets was based on the reported article with minor modifications, including three steps.^67^ First, 0.5 g of h-BN powder and 20 ml of benzyl benzoate were added into four sealed steel milling vials with 25 g steel balls that are 12.7 mm in diameter. The vials were filled with pure argon gas at a pressure of 200 kPa to avoid environmental contamination. The rotation speed of the four-station horizontal planetary mill was set at 350 rpm to generate enough shearing force to effectively exfoliate the h-BN powder. The milling time was set at 24 h. Secondly, the milled product was diluted with benzyl benzoate and sonicated at 100 W for 12 h to peel off the h-BN flakes more thoroughly. Finally, the modified h-BN was centrifuged at 3000 rpm for 20 min to remove the agglomerated large particles. The supernatant was collected and washed three times with absolute ethanol. The final product was filtrated and dried at 80 °C under vacuum for 12 h. The resulting nano-scale flakes were designated as modified boron nitride or MBN.

The surface morphology of the boron nitride before and after modification was prepared in the same way as the PBMG. It was diluted with distilled water to achieve the desired concentration, single drops of the diluted solution were deposited on a silicon wafer and placed in a vacuum for drying at room temperature for 24 h then examined with the SEM. The BN powder before and after modification was analyzed by Bruker AXS D8 Advance X-ray diffractometer at a scan range of 10–90°, and a scan rate of 6° min^−1^. The h-BN and MBN powder were also analyzed with a DXR Raman Microscope from Thermal Scientific Corporation. The laser wavelength was 532 nm.

### 2.6 Preparation of the composite samples

Initially the PBMG and MBN powder were dried in vacuum ovens at 40 °C and 80 °C, respectively, for 24 h. The weighed amount of PBMG and MBN were sequentially added to 80 g of epoxy, and the mixture was fairly dispersed for 15 min using a three-roll grinder to ensure that the fillers were evenly distributed in the epoxy. Then a hardener (68 g) and accelerator (1.6 g) were added to the mixture and stirred with a glass rod. Finally, the mixtures were degassed in a centrifugal deaerator for 10 min at a revolution speed of 3000 rpm, and a rotation speed of 900 rpm. The mixtures were poured into stainless steel molds slowly after degassing and cured at 90 °C for 1 h, then post-cured at 150 °C for 4 h to ensure complete cure of the composites. Samples were of EP/5 wt% PBMG with MBN contents of 1%, 3%, 5%, 7%, and 10% by weight. The size of the composite specimens in each sample was 80 mm long, 10 mm wide, and 4 mm thick.

### 2.7 Mechanical properties and failure analysis

Impact and flexure properties were obtained using China National Standards Testing Methods. The composite samples were conditioned at 23 ± 1 °C and 50 ± 5% relative humidity for 48 h before testing. The unnotched impact strength was determined according to the National Standard Testing Methods GB/T2571-1995 using a SANS ZBC-4 impact testing machine. The flexure properties were obtained according to Standard GB/T2570-1995 using a SANS CMT-6104 universal testing machine. Six replicate specimens of dimensions 80 mm × 10 mm × 4 mm were used in each of the impact and flexure tests. The fracture surface of failed specimens of the EP/PBMG/MBN composites samples were analyzed using SEM.

### 2.8 Thermal conductivity measurement of the composites

The thermal conductivities of the EP/PBMG/MBN composites were evaluated by TC 3000 Series Thermal Conductivity Apparatus (Xi'an Xiaxi Co. Ltd., China), based on transient hot-wire technique at room temperature according to ASTM C1113-99 (2004). Every specimen was measured for five times at room temperature and an average value was calculated.

## 3. Results and discussion

### 3.1 Preparation of PBMG toughening particles and modification of h-BN powder

#### 3.1.1 Preparation of PBMG toughening particles

A semi-continuous starvation-feeding method was used to prepare the toughening rubber-core PMMA-shell particles designated here as PBMG. The method has been widely used in the preparation of core–shell polymers.^65^ First, the deposition rate of monomers must be slightly lower than the reaction rate of the monomer to prevent secondary nucleation or heterostructure of the core–shell particles formed during the polymerization process. Second, the reaction activity of the monomers should be relatively high to ensure the effectiveness of the starvation-feeding method.

**Fig. 2:**
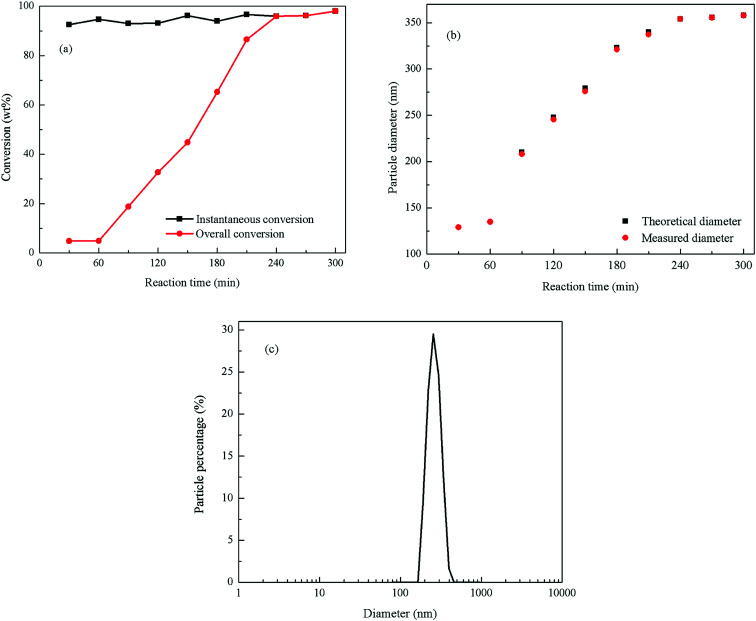
Emulsion polymerization of PBMG (a) monomer conversion *vs.* reaction time; (b) theoretical and measured diameter of latex particles *vs.* reaction time; and (c) particle size distribution and morphology of the final PBMG.

The monomer conversion *vs.* reaction time of the emulsion polymerization is shown in Fig. 2(a). It can be seen in this figure that the monomers instantaneous conversion rates are higher than 90%, and the final conversion rate is 97.45%. The total monomer conversion with the reaction time exhibited a linear growth mode, indicating that the polymerization process was relatively stable, and that the deposition rate of the monomers was appropriate in this reaction. During the stabilization stage (240–300 min), the instantaneous conversion of monomers coincided with the total conversion and the total conversion rate basically remained unchanged signaling that no more monomers were added to the reaction.

Fig. 2(b) shows the measured and theoretical particle sizes *vs.* the reaction time. The figure presents that the particle diameter was 130 nm at the end of the seed stage, and the final particle diameter was 358 nm. The measured particle size of latex particles was basically consistent with the theoretical particle size, which indicates that all the added monomers were polymerized on the surface of the original latex particle without secondary nucleation. Fig. 2(c) shows the distribution of the final particle size of the PBMG latex. The particle distribution index was 0.044, which means that the final particles size of the latex was uniform with a very narrow distribution range.

**Fig. 3:**
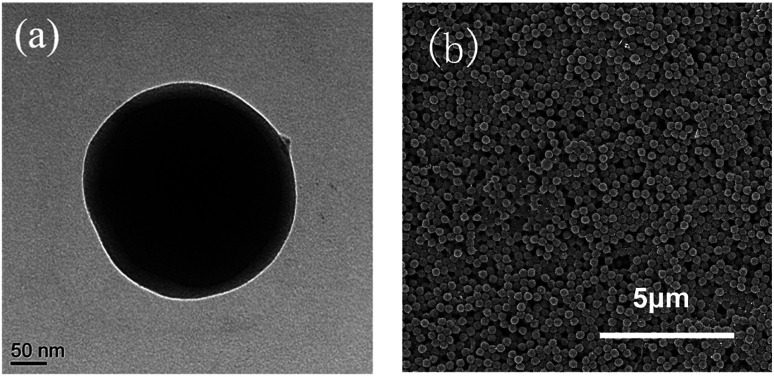
PBMG latex (a) TEM core–shell structure; (b) SEM particle distribution.

The PBMG latex was further analyzed by TEM and SEM. As shown in Fig. 3(a), the particles consisted of a dark core of poly(BA) and a brighter shell of poly(MMA), which clearly indicated that a core–shell structure of PBMG latex had been successfully constructed. The SEM image in Fig. 3(b) shows that the PBMG latex had a uniform distribution and consistent size.

#### 3.1.2 Exfoliation and modification of h-BN

**Fig. 4:**
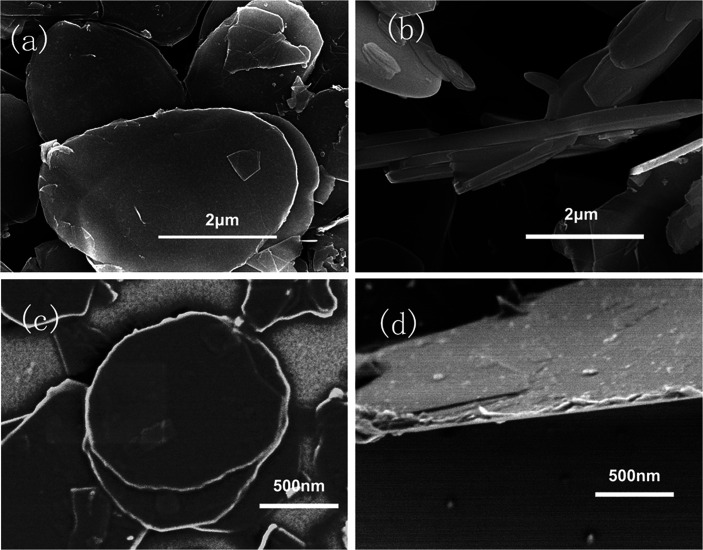
SEM images of h-BN and MBN (a) lateral size of h-BN, 3–5 μm; (b) thickness of h-BN, 300 nm; (c) lateral size of MBN, 1–2 μm; (d) thickness of MBN, 30 nm.

Boron nitride powder was used to improve the thermal conductivity of EP/PBMG matrix. To maximize its efficiency, the h-BN was modified by peeling the flakes into nanosheets and ensuring the exfoliation and well dispersion of the sheets in the composite. SEM images of h-BN before and after modification are shown in Fig. 4. Fig. 4(a) and (b) show that the bulk h-BN flakes have lateral dimensions of 3–5 μm and thickness of ∼300 nm. Fig. 4(c) and (d) show that the modified boron nitride (MBN) sheets have lateral dimensions of 1–2 μm and thickness of 30 nm indicating that the ball milling and sonication process were effective in peeling off thin layers of h-BN from bulk flakes and/or breaking the flakes into smaller pieces. It is worth noting that the edge of the MBN is curly and rough which could only be seen in the nanosheet samples. All observations demonstrated that exfoliated layers of boron nitride nanosheets were successfully obtained.

**Fig. 5:**
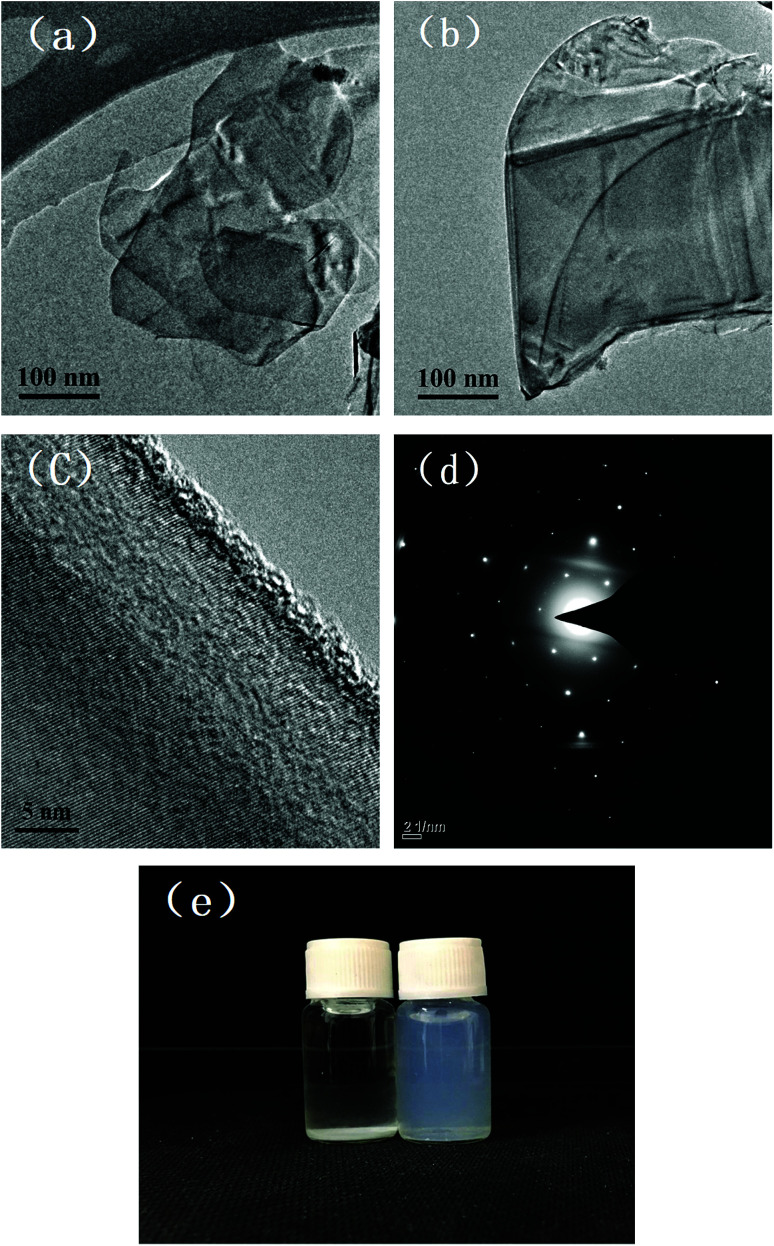
TEM images of MBN (a) & (b) low resolution; (c) HRTEM; (d) electron diffraction pattern; and (e) photos of h-BN and MBN dispersed in ethanol solvent for 24 h.

Thin specimens of MBN were prepared and analyzed under the TEM. The thin and transparent MBN nanosheets shown in Fig. 5(a) and (b) indicate the successful modification of h-BN. From the high resolution TEM images and selected area electron diffraction (SAED) images of MBN in Fig. 5(c) and (d), it can be seen that the MBN lattice fringes are still clear and continuous after the extremely high shear forces in the ball milling process. The SAED pattern is obtained from the TEM measurements. Fig. 5(d) is taken with an electron beam along [001] zone axis, perpendicular to the surface of the MBN nanosheets. It reveals the typical sixfold symmetry of h-BN, which indicates that the exfoliated MBN nanosheets retained the well-crystallized nature and structural integrity of h-BN.^68,69^Fig. 5(e) is a photograph of h-BN and MBN dispersed in ethanol solvent for 24 hours. As the peeled boron nitride nanosheets have less layers and lighter mass, they are evenly suspended in ethanol solvent without sedimentation; on the other hand, the unmodified boron nitride microplates settled at the bottom of the bottle due to the high density of the thicker layers.

**Fig. 6:**
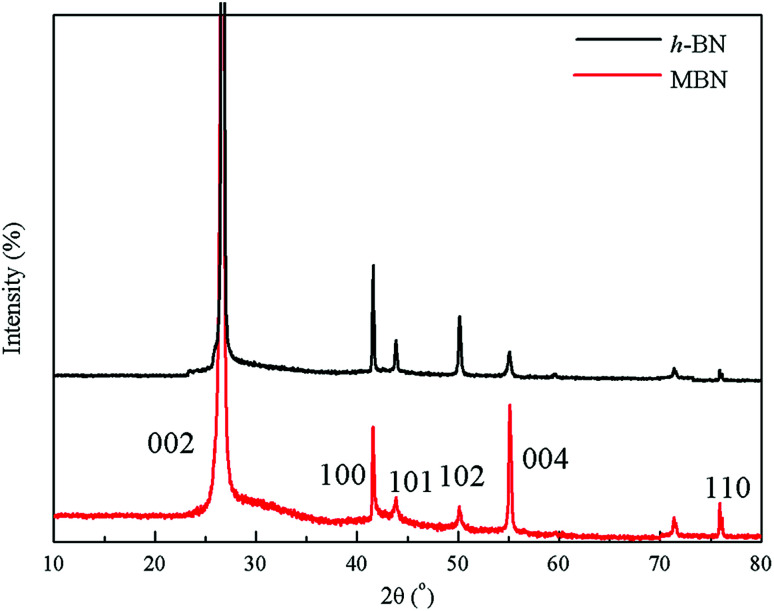
XRD patterns of h-BN and MBN.

The crystal structure and exfoliation of the MBN were characterized by X-ray diffraction. The XRD patterns shown in Fig. 6 indicate that almost all the peak positions of h-BN and MBN are matching. The diffraction peaks located at 26.72°, 41.63° and 55.14° of 2*θ* angle corresponded to the (002), (100) and (004) planes according to the standard spectrum of hexagonal boron nitride.^70^ Both X-ray diffraction patterns of h-BN and MBN were narrow and sharp, without new peaks. These results indicate that MBN nanosheets prepared by ball milling and sonication could well maintain the original crystal structure.

Based on these results, the (002) crystal plane along the *C* axis was the optimal orientation of the BN layered material.^10,71^ Therefore, the better the exfoliation of boron nitride, the more (002) crystal planes are exposed in the BN. Comparing the two diffraction patterns, the intensities of the (002) and (004) planes of the MBN are significantly enhanced and the intensities of diffraction peaks of other crystal planes are weakened, which indicates that more (002) planes were exposed during the modification process. Furthermore, according to the calculated result, the value of *I*_(004)_/*I*_(100)_ of MBN was 5 times higher than the original h-BN which fully proved that the MBN had been sufficiently exfoliated.^72^

**Fig. 7:**
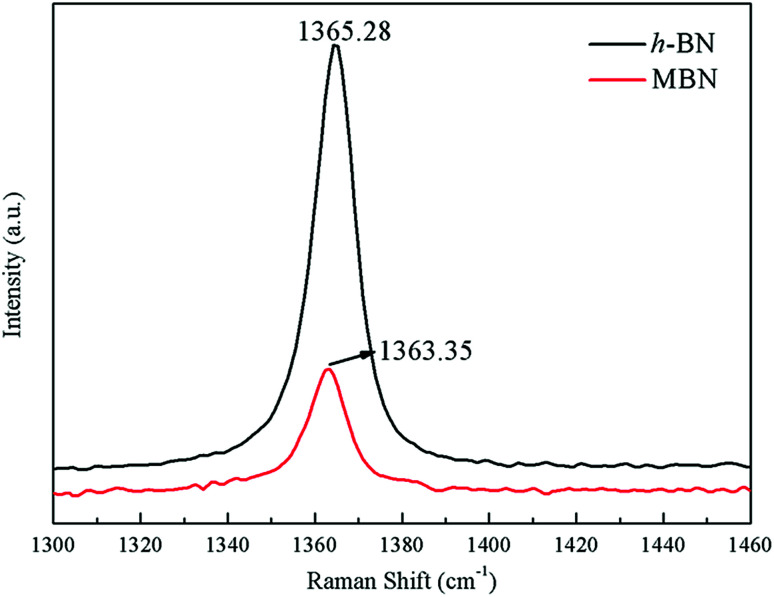
Raman spectra of h-BN and MBN.

The exfoliation of the MBN nanosheets was further verified using Raman microscopy, which was an effective method to characterize the structure and interlayer relationship of boron nitride.^73–75^Fig. 7 shows Raman spectra of h-BN and MBN. As can be seen from this figure, the characteristic peak position of h-BN and MBN were at 1365.28 cm^−1^ and 1363.35 cm^−1^, respectively, which attributed to the E_2g_ vibration mode of boron nitride material.^76,77^ Compared with h-BN, the Raman peak of the MBN produced a red shift of 1.93 cm^−1^ after exfoliation and the full width at half-maximum (FWHM) of the MBN nanosheets increased. This was because the stretching vibration between the boron nitride nanosheets and silicon substrate in the Raman test affected the E_2g_ vibration of MBN, and the nanolayer structure of MBN had produced photon limitations. These results indicated that the exfoliation could effectively reduce the thickness of BN powder.^78^ A similar phenomenon had been reported by Hou.^79^ In addition, the Raman peak intensity of MBN was significantly lower than that of h-BN, which may be explained by the widening of the spacing between the layers and the weakening of the interlaminar forces of boron nitride nanosheets.^80^ In short, Raman spectroscopy proved that the wet ball milling combined with ultrasonic method efficiently exfoliated the h-BN, as indicated by the TEM images and the XRD analysis above.

### 3.2 Improvement of impact toughness and flexure strength

**Fig. 8:**
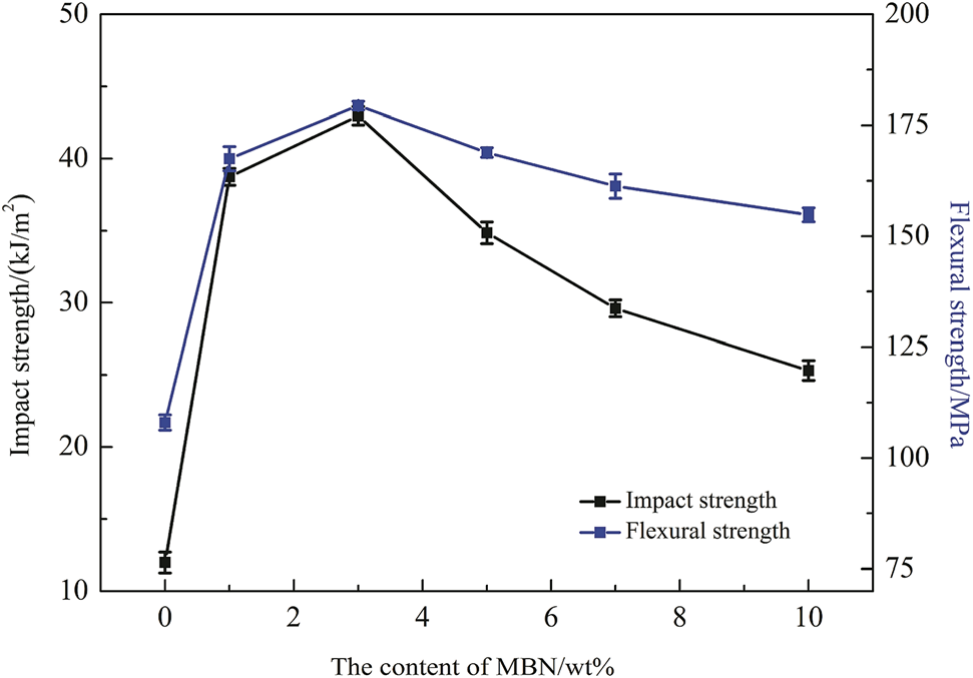
Impact and flexural strengths of epoxy composites with various MBN contents.

Before determining the change in thermal conductivity by the addition of MBN into the EP/PBMG blends, it was necessary to ensure that the addition of MBN did not significantly reduce the mechanical properties of the blended epoxy achieved with 5 wt% PBMG content. A study of the effect of the core–shell PBMG on the mechanical properties of epoxy showed that the impact and flexure strengths of the EP/PBMG increased with the PBMG content up to a certain weight fraction after which these properties started to decrease. The optimum PBMG content to obtain the maximum toughness and flexural strength was 5 wt%.^81^ At this content the impact and flexural strengths reached a maximum of 37.25 kJ m^−2^ and 168.4 MPa, respectively. The PBMG structure provided a PMMA shell which was compatible with the EP as the PBMG was functionalized with GMA on the outer shell. The epoxy groups of GMA on the surface of PBMG reacted with the epoxy resin during the curing process.^10,82^ In addition, at 5 wt% the PBMG was well-dispersed and evenly distributed in the EP matrix. The increased plastic deformation and energy absorption of the EP/PBMG were mainly due to the rubber core in the PBMG particles. The deformation mechanism is most likely due to stress-activated shear yielding initiated in the matrix, in the regions of high stress concentration close to the equators of the rubber particles. The deformation leads to the formation of cavitation within the rubber particles which in turn promotes shear-yielding and higher ductility.^81^ A similar study was conducted for the EP/PBMG/MBN composite in which the PBMG was kept at its optimum value of 5 wt% and the MBN content was increased from 0 to 10% by weight. Fig. 8 shows the variation of the impact strength and flexural strength of EP/PBMG/MBN composites with various MBN contents. It is shown in this figure that the impact and flexural strengths increase up to a certain amount of MBN content, then start to gradually decrease with additional content of MBN. The ability of the EP/PBMG to maintain its toughness and strength at low MBN content could be attributed to the high specific surface area and high surface activity of MBN, and a strong interaction between the MBN and epoxy matrix. In addition, the MBN may function as a layered reinforcement, hence increasing or maintaining the strength and toughness of the blended matrix. The highest impact and flexural strengths were achieved at 3 wt% MBN content. The impact strength at 3 wt% MBN was 43.0 kJ m^−2^ and the flexural strength was 179 MPa as shown in Fig. 8. The mechanical properties of the EP/PBMG/MBN composites started to gradually decrease after the MBN content exceeded 3 wt%. This is most likely due to the agglomeration of MBN at higher concentrations that lead to the formation of brittle regions with high stress concentrations. As shown in the next section, the thermal conductivity increased linearly with MBN content. The optimum strengths at a relatively high thermal conductivity were achieved with a composite consisting of 7 wt% MBN by weight. At this MBN content the impact strength of the EP/PBMG/MBN composites was 29.6 kJ m^−2^ and the flexural strength was 161 MPa. These values are lower than the strengths obtained at 3 wt% MBN content, however they are still 147% and 49.1%, respectively, higher than those of the neat epoxy. The major toughening mechanisms involve a suitable structured PBMG particle cavitation and/or debonding between the exfoliated BN layer and the EP, followed by massive shear yielding of the matrix.

**Fig. 9:**
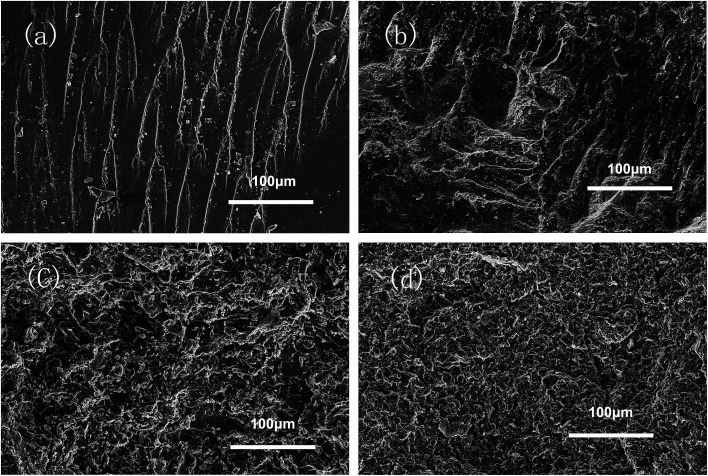
SEM images of the fracture surface of samples: (a) neat EP; (b) EP/PBMG/h-BN (3 wt%) composite; (c) EP/PBMG/MBN (3 wt%) composite; (d) EP/PBMG/MBN (10 wt%) composite.

In order to evaluate the interfacial interaction of h-BN and MBN with PBMG and EP matrix, the fractured surface of EP/PBMG/BN composites were investigated by SEM. Fig. 9(a) shows that the neat epoxy resin exhibits a relatively smooth fracture surface. The river pattern indicates a typical cleavage fracture, accounting for the low fracture toughness of the unfilled epoxy. In contrast, the EP/PBMG/BN composites show much rougher fracture surfaces indicating plastic deformation, as shown in Fig. 9(b) and (c). The increased surface roughness implies that the path of the crack tip is either arrested or deflected by the filler, making crack propagation more difficult.^83^ Large amount of micro cavities, ‘toughness dimples’ and macroscopically sized stress yielded zones can be observed, which indicate the development of localized shear-yielding plastic deformation under impact. Fig. 9(b) and (c) show that the composites exhibited plastic deformation and ductile failure at 3 wt% content of both h-BN and MBN. That could be due to the fact that the nanoscale BN may have stronger interfacial bond which can inhibit crack propagation or result in crack deflection, leading to higher energy absorption and tougher matrix.^84^ However, as shown in Fig. 9(d), with the increase of MBN content, agglomeration occurs gradually within the composite due to the van der Waals interactions between the adjacent MBN nanosheets, leading to some defects that may compromise the mechanical properties of the composites. In summary, the SEM images of fracture surfaces confirm that the incorporation of PBMG and exfoliated BN effectively improve the strength and toughness of EP/PBMG/MBN composites and illustrate the reasoning behind the relative decrease in these properties with higher MBN content.

### 3.3 Improvement of thermal conductivity

**Fig. 10:**
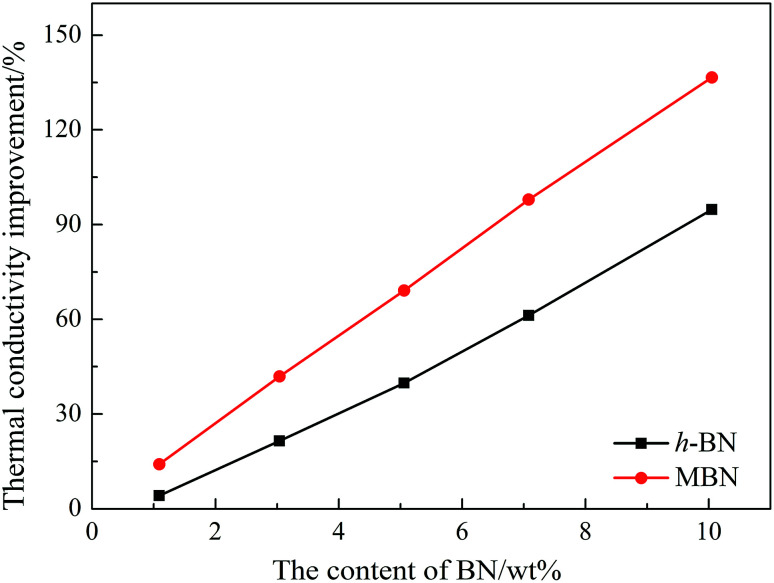
Thermal conductivity improvement of EP/PBMG/BN composites with different BN contents.

The thermal conductivity of the neat epoxy, the EP/PBMG blend, and the EP/PBMG/MBN composites with various MBN contents were investigated. In addition, the thermal conductivity of the EP/PBMG/h-BN composites were also investigated to clarify the importance of modifying the original h-BN. The thermal conductivity of the neat epoxy was determined as 0.191 W (m^−1^ K^−1^), and that of the EP/PBMG was measured at 0.192 W (m^−1^ K^−1^), indicating that the addition of core–shell particles did not affect the thermal conductivity of the epoxy, as shown in Fig. 10 and Table 1 below. The addition of BN leads to an increase in thermal conductivity and the percent increase in thermal conductivity, determined as the thermal conductivity improvement ratio in eqn (4) below, is shown in Fig. 10.

4 Thermal conductivity improvement ratio = [(thermal conductivity of the composite − thermal conductivity of the epoxy)/thermal conductivity of the epoxy] × 100

**Table 1:** Thermal conductivity of epoxy resin and its composites

Filler	Thermal conductivity with different BN contents (W (m^−1^ K^−1^))						
	EP	EP/PBMG	1 wt%	3 wt%	5 wt%	7 wt%	10 wt%
h-BN	0.191	0.192	0.199	0.232	0.267	0.308	0.372
MBN			0.218	0.271	0.323	0.378	0.452

It is shown in Fig. 10 that the thermal conductivity of the EP/PBMG/BN composites increases with the addition of both h-BN and MBN. More importantly, the graphs show that the addition of MBN is more effective in increasing the thermal conductivity of the composites than the h-BN. The high aspect ratio of the MBN nanosheets is critical in increasing the thermal conductivity improvement ratio shown in Fig. 10. The high aspect ratio of the MBN nanosheets leads to the formation of effective networks of conductive pathways that in turn lead to higher thermal conductivity.^76^ In addition, the high surface activity of the MBN nanosheets and their strong bond with the epoxy matrix provide stable and highly efficient conductive networks, further enhancing the thermal conductivity of EP/PBMG/MBN composites.

Table 1 shows that at 10 wt% the thermal conductivity of the MBN-filled composite is 0.452 W (m^−1^ K^−1^), while that of the EP/PBMG/h-BN composite is 0.372 W (m^−1^ K^−1^). These values are 1.37 and 0.95 times higher, respectively, than that of the neat epoxy matrix. In other words, at 10 wt% MBN, the thermal conductivity of the composite increased by 137%.

## 4. Conclusions

Poly(*n*-butyl acrylate)/poly(methyl methacrylate-*co*-glycidyl methacrylate) (PBMG) core–shell particles were synthesized by seed emulsion polymerization and added to epoxy resin to improve its toughness and strength properties. Modified boron nitride (MBN) nano-flakes were prepared by ball milling and sonication and added to the EP/PBMG blend to improve the thermal conductivity of the composite. The thermal conductivity of the EP/PBMG/MBN composite increased linearly with MBN content and reached a maximum value of 0.452 W (m^−1^ K^−1^), at MBN content of 10% by weight. The highest impact and flexural strengths were achieved at 5 wt% PBMG and 3 wt% MBN, but at 3 wt% MBN content the thermal conductivity was only 0.271 W (m^−1^ K^−1^). Therefore to get a good balance between the mechanical properties and the thermal conductivity, a 7 wt% MBN content is recommended. At this content the thermal conductivity is increased by 98% compared with pristine EP, the impact strength of the EP/PBMG matrix is increased by 147% from 12.0 kJ m^−2^ to 29.6 kJ m^−2^, and the flexure strength is increased by 49.1% from 108 MPa to 161 MPa. Material scientists and engineers can determine the appropriate MBN content within these limits to achieve desired toughness and thermal conductivity for specific applications. With the improved toughness and thermal conductivity, the EP/PBMG/MBN composite has high potential for applications in industries in which these properties are critical.

## Conflicts of interest

There are no conflicts to declare.

## Supplementary Material
